# RP-DETR: end-to-end rice pests detection using a transformer

**DOI:** 10.1186/s13007-025-01381-w

**Published:** 2025-05-17

**Authors:** Jinsheng Wang, Tao Wang, Qin Xu, Lu Gao, Guosong Gu, Liangquan Jia, Chong Yao

**Affiliations:** 1 School of Information Engineering, Huzhou University, Huzhou, 313000 China; 2 School of Information Science and Engineering, Jiaxing University, Jiaxing, 314001 China; 3 Huzhou Central Hospital, Huzhou, 313000 China

**Keywords:** Rice pest, Deep learning, RepPConv-block

## Abstract

Pest infestations in rice crops greatly affect yield and quality, making early detection essential. As most rice pests affect leaves and rhizomes, visual inspection of rice for pests is becoming increasingly important. In precision agriculture, fast and accurate automatic pest identification is essential. To tackle this issue, multiple models utilizing computer vision and deep learning have been applied. Owing to its high efficiency, deep learning is now the favored approach for detecting plant pests. In this regard, the paper introduces an effective rice pest detection framework utilizing the Transformer architecture, designed to capture long-range features. The paper enhances the original model by adding the self-developed RepPConv-block to reduce the problem of information redundancy in feature extraction in the model backbone and to a certain extent reduce the model parameters. The original model’s CCFM structure is enhanced by integrating the Gold-YOLO neck, improving its ability to fuse multi-scale features. Additionally, the MPDIoU-based loss function enhances the model’s detection performance. Using the self-constructed high-quality rice pest dataset, the model achieves higher identification accuracy while reducing the number of parameters. The proposed RP18-DETR and RP34-DETR models reduce parameters by 16.5% and 25.8%, respectively, compared to the original RT18-DETR and RT34-DETR models. With a threshold of 0.5, the average accuracy calculated is 1.2% higher for RP18-DETR than for RT18-DETR.

## Introduction

Pest damage to rice is one of the main reasons for the threat to the safe production of rice grain. Due to the wide area of rice cultivation worldwide, the large regional differences, the complex changes in climatic conditions, and the characteristics of pests, which are large in number, diverse in species, and develop rapidly, pest control in rice poses a huge challenge. Common rice pests and diseases mainly include “three insects and three diseases”: rice planthopper, leaf roller, stem borer, rice false smut, rice blast, and sheath blight [1, 2]. According to statistics, insect pests reduce rice yields by more than 5% in China every year [1]. Therefore, timely and accurate grasp of the types of pests and the areas covered during different growth periods of rice can not only quickly take targeted control measures to reduce the economic losses caused by pests to rice production, but also avoid the pollution of the ecological environment caused by blind use of pesticides. Traditional rice pest monitoring mainly relies on experts to observe the external characteristics of pests on the surface of rice leaves or rhizomes through sampling. This subjective manual identification method is closely related to the experience of the expert, and the identification process is cumbersome and time-consuming, which is difficult to meet the application requirements of large-scale and rapid pest monitoring in actual production [3]. Rice pests seriously endanger rice cultivation, affect rice growth and reduce grain yields. In view of the inefficient and costly manual detection, which cannot be carried out in real time on a large scale, this paper conducts an in-depth study on the precise identification of rice pests, with a view to carrying out targeted pest control, reducing detection costs and increasing rice yields.

In recent years, more and more researchers have used deep learning methods for plant pest and disease research [4–11], while automatic identification of rice pests and diseases based on machine vision has attracted widespread attention from scientific researchers [12–14]. The basic principle of this type of method is to first construct a model for representing the visual features of an image, then train this model on a labeled training dataset to determine the parameters of the algorithmic model, and finally verify the performance of the algorithm on a test dataset. From the perspective of visual feature representation, machine vision-based pest identification methods for rice can be roughly divided into two categories: handcrafted feature-based [15–18] and deep learning feature-based [21–23]. Manual features refer to the construction of multiple visual feature expression models based on the pixel distribution characteristics of the image, such as color histograms [17], local binary patterns [18], etc. Handcrafted features Due to the simple representation of features, which only express shallow levels of vision, in practical applications, visual expression is performed using a combination of multiple handcrafted features. For example, MA Pengpeng et al. [15] studied the impact of global visual features, local visual features, and the fusion of the two on the recognition results of target pests in rice, in view of issues such as image feature selection and sample imbalance. They divided rice lamp-trapped insects into large and small insects based on the body size of five target pests. BAO Wenxia et al. [17] used the sliding window method to extract the HSV color features and SILTP texture features of rice pest images, and used the deep semantic segmentation U-Net network to remove complex backgrounds. An elliptical metric model with enhanced data discrimination was introduced to extract spatial structures and semantic features of rice pest images, while capturing potential relationships among image features. YANG Ying et al. [18] proposed a method that combines weighted fusion of directional gradient histograms and local binary pattern features to extract visual features from rice pest and disease images, aiming to address the challenges of large sample requirements and high processing equipment demands. Deep learning features refer to the visual feature expression established for an image through a deep neural network model [19]. The widely used neural network model is the deep convolutional neural network, which stacks convolutional layers, pooling layers, activation layers, and optimally fully connected layers for image classification [20]. The significant advantage of this model is its strong feature expression ability. The network model can extract local shallow visual features through the underlying convolutional layer, and rich global semantic features through the high-level convolutional layer. Unlike handcrafted features, model feature expressions learn discriminative features from image pixels. Their feature expressions are closely related to the training data, while handcrafted feature expressions are not related to the training data. For example, Huang Shuangping et al. [21] constructed a deep neural network by stacking Inception modules to establish the feature expression of rice panicle blast images. They employed multi-scale convolutional kernels to extract panicle blast spot features at various scales, applied cascade fusion, and introduced a detection method based on the GoogLeNet deep convolutional neural network. TAN Yun-lan et al. [22] used a deep convolutional neural network model to address the problem of the complex and changeable characteristics of rice image disease boundaries. A dataset augmentation technique was used, and a parameter fine-tuning method was used to optimize the network, achieving image recognition of the eight common types of rice diseases captured in natural scenes. Fan Chunquan et al. [23] developed a comprehensive rice pest recognition dataset with over 20,000 images of 16 pest species to improve identification performance, addressing issues caused by limited data and insufficient diversity. ResNet50 served as the backbone network for designing and validating the rice pest recognition model. Fengchang et al. [24] fused Swin Transformer with DETR, using Swin Transformer’s good feature extraction ability for small targets combined with DETR model’s ability to detect large targets. Using the latest SwiGLU activation function improved the algorithm model’s recognition ability for target images and enabled it to detect and process targets of different scales and shapes.

The studies mentioned above share certain limitations. Primarily, training these network models depends on a large volume of precise image data. Only by relying on a large and accurate data set can the effect of model training be guaranteed. Second, the current model has problems with misidentifying backgrounds, missing detections and false detections in the study of rice pests, and its real-time detection ability and practicality are weak.

Inspired by the results of the DETR [25] model and in response to the above problems, this study proposes a new detection algorithm based on Transformer [26] for identifying seven rice pests. This method improves the initial RT-DETR model by fine-tuning it on a self-built multi-source fusion dataset of 7868 rice pest data.

The key contributions of this paper are:

1. Considering the limitations of existing rice pest datasets with missing and incomplete samples, this paper constructs a dataset tailored for deep learning model training by analyzing the characteristics of rice pests in their natural environment. It includes seven common types of pests. This dataset serves as a strong foundation for the deep learning algorithm in this study, guaranteeing the robustness and effectiveness of the pest detection model.
2. Given the RT-DETR model’s real-time and convenient application in pest detection, this paper introduces a new RepPConv-block structure to reduce model parameters, minimize information redundancy in feature extraction, and enhance real-time detection performance.
3. To address false positives and false negatives in the RT-DETR model caused by complex plant backgrounds, multiple disease categories, and high disease similarity, this paper enhances the CCFM module by integrating the Gold-YOLO-Neck structure. This improvement strengthens feature fusion in the network decoder, allowing the model to capture richer multi-scale semantic information, focus more on pest targets, reduce background sensitivity, minimize missed detections, and enhance both practicality and adaptability.
4. To improve positioning accuracy for rice pests of varying shapes and sizes, this paper introduces the MPDIoU target regression loss function, enhancing the model’s ability to precisely regress target boxes and improving overall detection accuracy.

## Materials and methods

### Dataset

The image data were collected using various mobile photography devices to capture rice fields in Huzhou City, Zhejiang Province, supplemented with online images and select rice pest images from the IP102 dataset. Through continuous observation in different seasons, the team obtained images of the real scene covering the entire growth cycle of rice. These images, captured under natural lighting in multiple greenhouses, encompass diverse environmental factors, including background variations, complex site conditions, and different lighting settings. This diversity enhances the dataset’s richness, improving the model’s robustness and generalizability.

The IP102 [27] dataset contains more than 75,000 images covering 102 categories, showing a long-tailed distribution in nature. This dataset was collected through a wide range of the Internet of Things, including sources such as ImageNet [28], COCO [29], graphic search engines, and multiple professional entomological science websites. The IP102 dataset contains 8471 images of rice pests covering 14 categories. Considering the model performance and the common threat of rice pests, seven pests in IP102 were selected as the detection objects in this experiment.

**Fig. 1:**
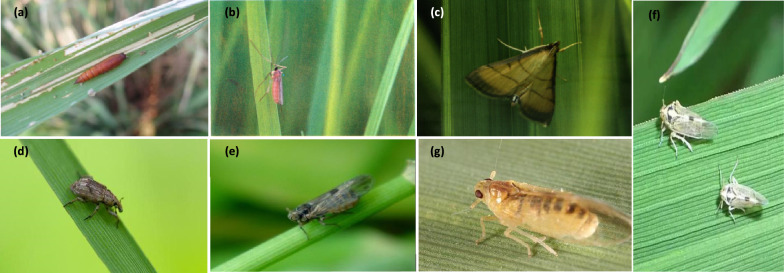
**a** Asiatic rice borer **b** rice gall midge **c** rice leaf roller **d** rice water weevil **e** small brown plant hopper **f** white backed plant hopper **g** yellow rice borer

Based on this, the experiment obtained more diverse image data through offline image collection and online image collection. Several members of the research team manually selected available image data and uniformly processed it to 640 × 640 pixels, ultimately creating a high-quality rice pest data set containing 7 categories. Table 1 lists the amount of data for different types of pests in this experiment, and Fig. 1 shows a sample image of the data for this experiment.

**Table 1:** Dataset of rice pests

Category	Training set	Val set	Test set	Summary
Asiatic rice borer	823	283	104	1210
Rice gall midge	544	167	77	788
Rice leaf roller	761	189	97	1047
Rice water weevil	681	234	96	1011
Small brown plant hopper	933	248	121	1302
White backed plant hopper	1078	333	140	1551
Yellow rice borer	649	208	92	949
Summary	5469	1672	727	7868

**Fig. 2:**
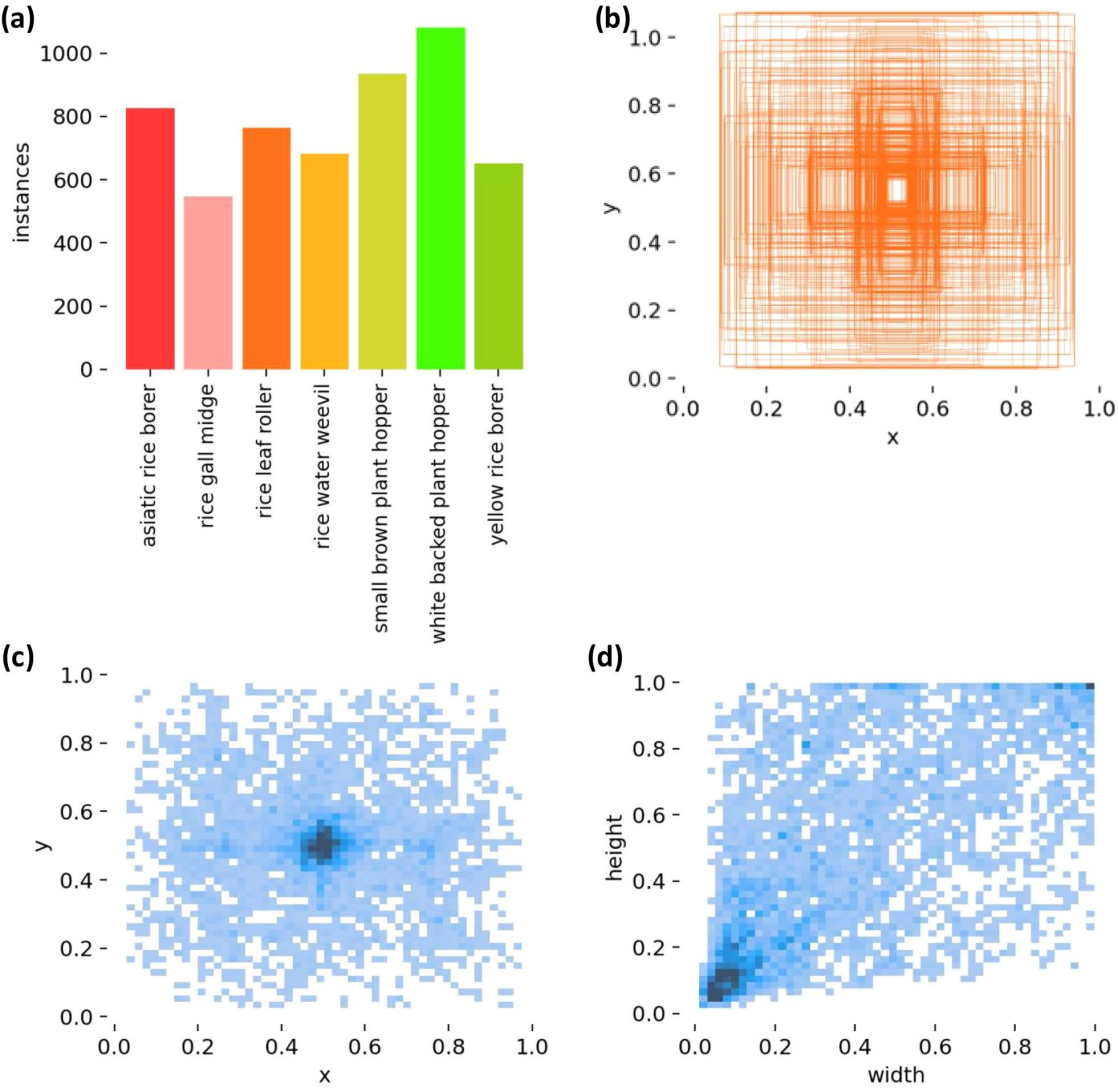
**a** Distribution of the number of pests in the dataset **b** Size and position of the anchor frame in the dataset **c** Position of the anchor frame in the dataset **d** Size of the anchor frame in the dataset

Figure 2a presents the data distribution of the rice pest dataset. The bar chart in the upper left highlights the seven main pest categories along with their respective sample counts. The X-axis represents specific pest types, including rice leaf roller, asiatic rice borer, rice gall midge, small brown plant hopper, rice leaf roller, yellow rice borer, rice water weevil, and white backed plant hopper. The Y-axis represents the sample count for each category. It can be clearly seen that yellow rice borer has the largest number, and its image samples are easy to collect, which to some extent indicates that it is more likely to appear in rice. The rice gall midge has the smallest sample size, which to some extent indicates that it is less likely to appear in rice pests. Other pests are more evenly distributed in the dataset. Figure 2b illustrates the distribution of anchor box positions and sizes within the image. The figure clearly shows that most anchor boxes in the dataset are centrally located, relatively small, and less frequent in other areas. Figure 2c displays the anchor box distribution in the image. The figure indicates that most anchor boxes are clustered around the center. Figure 2d presents the distribution of anchor box sizes. The figure reveals that most anchor frames are relatively small, reflecting their precision within the dataset.

### The proposed RP-DETR network structure

The field of Real-Time Object Detection has long been dominated by the YOLO series of models [30]. However, YOLO relies on non-maximum suppression (NMS) technology when detecting objects. NMS is a post-processing technique in object detection that eliminates highly overlapping detection boxes generated by the model. The technology includes two key hyperparameters: a confidence threshold and an intersection over union (IoU) threshold. Detection boxes below the confidence threshold are discarded, and if the IoU between two boxes exceeds the set limit, the one with lower confidence is eliminated. This process will be repeated until all the detection boxes for all categories have been processed. The NMS algorithm’s execution time depends on the number of predicted boxes and threshold settings, impacting detection speed and model robustness. In recent years, vision transformers have received more attention in the application of machine vision [31, 32].

RT-DETR [33] is a new real-time end-to-end object detector that not only surpasses current real-time detectors in accuracy and speed, but also does not require post-processing, thereby avoiding inference speed delays and ensuring stable results. Inspired by RT-DETR, the paper designs a Transformer detection model RP-DETR for rice pest detection.

**Fig. 3:**
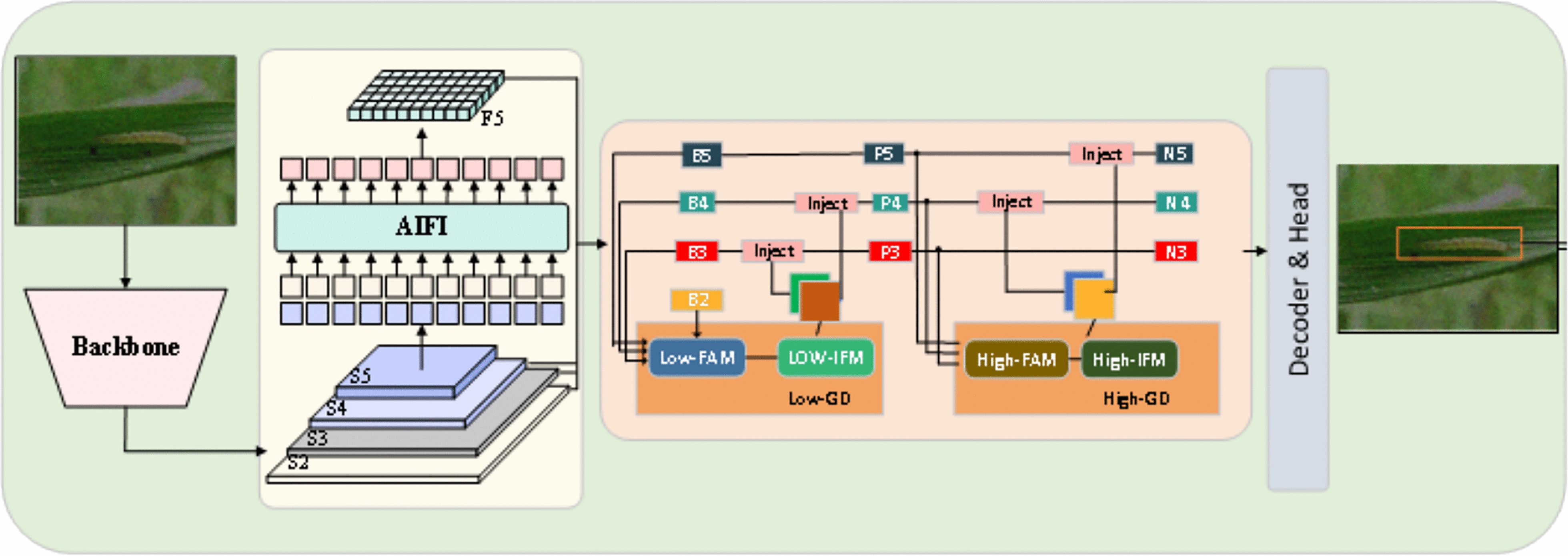
Schematic diagram of the RP-DETR Module

The RP-DETR model is innovative in its structural design. This experiment introduces the RepPConv-block to enhance the RT-DETR backbone, reducing parameters, minimizing feature extraction redundancy, optimizing throughput, and improving memory access efficiency. Second, the Gather-and-Distribute mechanism (GD) [34] is used to improve the original CCFM module as the decoder structure of RP-DETR. These innovative designs make the RP-DETR excel at the task of rice pest detection, with higher detection accuracy and efficiency. Figure 3 illustrates the RP-DETR network structure designed in this study.

#### Structure of the RepPconv-block

In this experiment, the RT-DETR model with ResNet18 [35] as the backbone network (hereinafter referred to as RT18) is used as the benchmark model. ResNet is known for its powerful feature extraction capabilities, thanks mainly to the introduction of residual networks. The residual network simplifies learning by transforming the mapping from X to Y into the difference Y-X, then adding the learned residual to the original output. While the residual structure improves feature extraction, it also adds numerous parameters and increases memory usage due to residual connections. To reduce parameters, lower computational complexity, and enhance inference speed in the RT-DETR network, this experiment introduces a novel residual structure block, the RepPConv-block.

Depthwise Separable Convolutions (DWConv) and Grouped Convolutions (GConv) are widely used to extract spatial features in some common network models, such as MobileNet [36], ShuffleNet [37] and GhostNet [38]. Depthwise Separable Convolution have the advantage of reducing the number of parameters, but replacing 2D convolutions with Depthwise Separable Convolutions may result in a suboptimal model with reduced model performance. In addition, Depthwise Separable Convolutions has high memory access requirements, which results in slow calculations on the GPU. Although the FLOPs are low, the latency is high. Grouped Convolutions reduces parameters but may cause global channel information loss due to limited interaction between groups. Reducing parameters and FLOPs often negatively impacts convolution operators due to increased memory access.

**Fig. 4:**
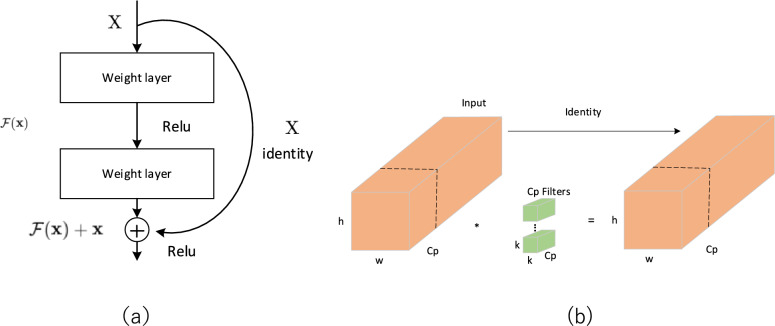
**a** Resnet block structural **b** Pconv block structure

Inspired by the FasterNet [39] network, this paper designs a brand new RepPConv-block to improve the residual block of ResNet. The Partial Convolution (PConv) [39] module in FasterNet is a convolution operator that can reduce computational redundancy and memory access. Figure 4a shows the structural principle of the Resnet block. Figure 4b illustrates the operation of PConv: it performs a standard convolution on a subset of the input channels for spatial feature extraction, while leaving the remaining channels unaffected. For sequential or typical memory access, the first or last consecutive channel is used as a representative of the full profile. It is assumed, without loss of generality, that the input and output feature maps contain the same number of channels.

Pconv has a good effect on network parameters and the degree of complexity of calculations. In order to reduce memory usage at the same time, this experiment further introduces the idea of structural reparameterization, using RepConv to reparameterize the model. Re-parameterizable Convolution (RepConv) [40] is a convolutional layer that uses structural reparameterization techniques. It has the ability to integrate multiple computational modules into a single unit during the inference phase, enhancing both the model’s efficiency and performance. RepConv’s core concept is to utilize multi-branch convolutional layers during training, and then reparameterize the branch parameters into the main branch during inference, which helps to decrease computational load and memory usage. The fundamental concept is illustrated in the following equation.

1 $$\begin{gathered} \begin{array}{*{20}c} {\hat{x}_{i} = \gamma \cdot \frac{{x_{i} - \mu }}{{\sqrt {\sigma^{2} + \varepsilon } }} + \beta } \\ { = \frac{\gamma }{{\sqrt {\sigma^{2} + \varepsilon } }} \cdot x_{i} + \left( {\beta - \frac{\gamma \cdot \mu }{{\sqrt {\sigma^{2} + \varepsilon } }}} \right)} \\ \end{array} \hfill \\ {\text{can be viewed in the form}} y = wx + b , \,{\text{where}} \,w_{BN} = \frac{\gamma }{{\sqrt {\sigma^{2} + \varepsilon } }},b_{BN} = \beta - \frac{\gamma \cdot \mu }{{\sqrt {\sigma^{2} + \varepsilon } }} \hfill \\ \end{gathered}$$

2 $$\begin{array}{*{20}c} {\hat{x} = w_{BN} \cdot \left( {w_{conv} \cdot x + b_{conv} } \right) + b_{BN} } \\ { = \left( {w_{BN} \cdot w_{conv} } \right) \cdot x + \left( {w_{BN} \cdot b_{conv} + b_{BN} } \right)} \\ \end{array}$$

3 $$\left\{ {\begin{array}{*{20}c} {w = w_{BN} \cdot w_{conv} } \\ {b = w_{BN} \cdot b_{conv} + b_{BN} } \\ \end{array} } \right.$$

Equations (1) to (3) describe the process of transforming the Batch Normalization (BN) layer into a linear transformation, which is then merged with the convolutional layer (Conv) to simplify the computational complexity. The parameters $\gamma$ and $\beta$ are learnable scaling and offset values, while $\mu$ and $\sigma^{2}$ represent the mean and variance, respectively. Additionally, ϵ is a small constant used for numerical stability.

The addition of the RepPConv-block maintains strong feature extraction capabilities for the model as a whole, while significantly reducing the number of parameters and memory footprint, thereby improving inference speed and overall performance. This will improve the real-time nature of rice pest detection and the effectiveness of the model in practical applications.

#### Gather-and-distribute structure

Multiscale feature fusion is a technique commonly used in object detection models to improve the model’s ability to detect objects of different sizes [41]. By combining features from different layers of the network, this technology can capture information from coarse to fine scales. Low-level features usually contain more details about small objects, while high-level features capture the semantic information of large objects. Multi-scale feature fusion improves the model’s ability to represent data by combining features from different levels, allowing it to better detect and localize objects of various sizes in the image.

The traditional target detection framework employs the FPN structure as the neck, merging high-level and low-level feature maps via up sampling. However, it can only fully combine features from adjacent layers, with information from other layers being indirectly transferred, potentially causing data loss. The multi-scale information transmission in the CCFM module of the RT-DETR network also has the same problem. To solve this problem, the paper draws on the new neck structure in Golod-YOLO [34]. This module introduces a mechanism for Gather-and-Distribute (GD mechanism), which collects and fuses information at all levels through unified modules and distributes it to different levels, thereby avoiding information loss, enhancing the ability of partial information fusion, and not increasing significant latency. The collection and distribution process involves three core modules: the Information Fusion Module (IFM), the Information Injector (Inject), and the Feature Alignment Module (FAM). During the collection process, FAM gathers and aligns features from each layer, while IFM merges these aligned features to create global information. The fused global information is transmitted to each level via the injection module, while a simple attention operation boosts the branch’s detection capability. To improve the model’s capability in detecting objects of various sizes, two branches are introduced: a High-Gathering and Distribution (High-GD) branch and a Low-Gathering and Distribution (Low-GD) branch. These branches are tasked with extracting and combining large and small feature maps, enhancing the model’s adaptability.

**Fig. 5:**
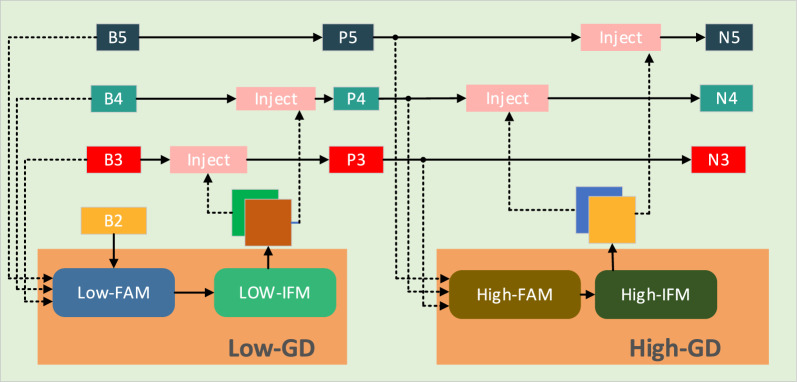
Gather-and-Distribute Schematic

In the RP-DETR network, paper discard the CCFM module in RT-DETR and adopt the Gather-and-Distribute structure. Figure 5 shows a schematic diagram of the Gather-and-Distribute structure. This enhancement refines the model’s hierarchical feature representation and broadens its sensory field. By integrating features from various scales, the model can capture a broader spectrum of contextual information, enhancing both the accuracy and reliability of target detection. This structure is particularly important as it helps preserve more semantic feature information at various levels, reducing the loss of semantic details.

#### MPDIoU loss function

Bounding box regression (BBR) is a crucial technology in object detection and instance segmentation, playing a key role in accurately positioning objects. While BBR excels in positioning accuracy, the current bounding box regression loss function has limitations in optimization, especially when there is a large difference between the predicted and ground truth box dimensions while keeping the aspect ratio constant. To address this challenge, Ma et al. [42] propose an innovative loss function based on the minimum point distance (MPDIoU), which aims to directly optimize the distance between the corners of the predicted box and the real annotated box. This method simplifies and combines all the key factors from the existing loss function, such as non-overlapping areas, overlapping areas, width-height differences, and center point distances, while also significantly improving computational efficiency.

The Minimum Point Distance Intersection over Union (MPDIoU) introduces a novel constraint mechanism that enhances bounding box regression performance by overcoming the limitations of the traditional IoU loss function. A key advantage is its ability to deliver useful gradient information, even in scenarios with little or no overlap. Traditional IoU-based metrics (such as GIoU [43], DIoU [44] and CIoU [44]) have difficulty optimizing bounding boxes when the intersection area is small, because the IoU value tends to approach zero at this time. MPDIoU, in contrast, offers a clear optimization path even when there is no overlap by introducing a predicted box and calculating the Euclidean distance between the top-left and bottom-right corners of the ground truth box. This ability makes it particularly effective when dealing with complex targeting problems.

MPDIoU not only remains highly robust with low overlap, but it also handles bounding boxes with the same aspect ratio but varying sizes effectively. Although CIoU includes a relative aspect ratio penalty term, it often fails to account for the true geometric differences when the predicted and ground truth boxes have the same aspect ratio. MPDIoU enhances the model’s capability to align bounding boxes by minimizing the absolute distance between the predicted box and the actual corner point of the true box, regardless of the size or aspect ratio similarity, thus allowing the loss function to better capture the true geometric deviation. Therefore, MPDIoU is very effective when dealing with detection tasks of multiple scale targets in complex scenes.

Another significant advantage of MPDIoU is the simplicity of its calculation. Unlike the CIoU and other advanced IoU variants, which penalize aspect ratios using complex operations such as arctangents, the MPDIoU relies only on basic geometric calculations such as distance and area calculations. This simplified calculation process reduces the calculation overhead, making it ideal for real-time applications or scenarios with limited computing resources. Moreover, this simplified form does not sacrifice performance. On the contrary, it further optimizes training efficiency through more direct measurement of degrees.

**Fig. 6:**
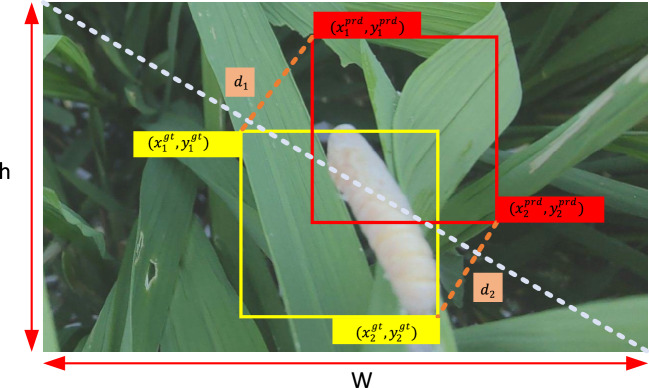
MPDIoU schematic

MPDIoU also effectively combines essential factors in bounding box regression, such as center point distance, overlap, variations in width and height, and non-overlap regions. By integrating these factors into one cohesive metric, MPDIoU provides a more precise alignment between the predicted and actual boxes. Moreover, this integration streamlines the implementation process, minimizes potential errors, and enhances the model’s overall performance. Figure 6 presents a diagram illustrating the MPDIoU.

Figure 6 visually demonstrates the geometric relationship between the rectangular frames in the MPDIoU method and the core idea of the calculation. The figure contains two rectangular boxes, of which the yellow box represents the ground truth. The coordinates of the vertices in the bottom-right corner and top-left corners are $\left( {x_{2}^{{{\text{gt}}}} ,y_{2}^{{{\text{gt}}}} } \right)$ and $\left( {x_{1}^{{{\text{gt}}}} ,y_{1}^{{{\text{gt}}}} } \right)$ respectively; the red frame is the prediction frame, and its corresponding vertex coordinates are $\left( {x_{1}^{{{\text{prd}}}} ,y_{1}^{{{\text{prd}}}} } \right)$ and $\left( {x_{2}^{{{\text{prd}}}} ,y_{2}^{{{\text{prd}}}} } \right)$ respectively.

4 $$d_{1}^{2} = \left( {x_{1}^{{\text{B}}} - x_{1}^{{\text{A}}} } \right)^{2} + \left( {y_{1}^{{\text{B}}} - y_{1}^{{\text{A}}} } \right)^{2}$$

5 $$d_{2}^{2} = \left( {x_{2}^{{\text{B}}} - x_{2}^{{\text{A}}} } \right)^{2} + \left( {y_{2}^{{\text{B}}} - y_{2}^{{\text{A}}} } \right)^{2}$$

6 $${\text{MPDIoU}} = \frac{A \cap B}{{A \cup B}} - \frac{{d_{1}^{2} }}{{w^{2} + h^{2} }} - \frac{{d_{2}^{2} }}{{w^{2} + h^{2} }}$$

In Eqs. 4–6, IoU represents the standard intersection over union, measuring the overlap between the predicted and actual frames. However, IoU fails to offer useful gradient information when the overlap between the predicted and actual boxes is minimal or nonexistent. For this purpose, MPDIoU introduces two additional distance terms: $d_{1}^{2}$ and $d_{2}^{2}$, which represent the Euclidean distance between the top-left and bottom-right corners of the predicted box and the ground truth box, respectively. The distances are normalized with a scaling factor to adjust for images of varying resolutions, ensuring that the distance value’s contribution is not influenced by the image’s width and height. The MPDIoU reference enhances the RT-DETR rice pest detection model’s predictive performance.

### Model evaluation criteria

In order to effectively evaluate the application of the RP-DETR network model to rice pests, the F1 score, Precision (P), mAP (mean of Average Precision), and Recall (R) are selected as the main evaluation metrics for this study [45]. In this experiment, the mAP evaluation metric uses the IoU threshold of 0.5 for mAP@0.5as another evaluation metric for this study [46]. In this experiment, the mAP@0.5 metric, which applies an IoU threshold of 0.5, is used as an extra evaluation criterion. The formulas for calculating precision, recall, F1 score, and mAP can be found in Eqs. 7, 8, 9, and 10, respectively.

7 $$\begin{array}{*{20}c} {Precision = \frac{TP}{{TP + FP}}} \\ \end{array}$$

8 $$\begin{array}{*{20}c} {recall = \frac{TP}{{TP + FN}}} \\ \end{array}$$

9 $$\begin{array}{*{20}c} {F1 = \frac{2TP}{{2TP + FN + FP}}} \\ \end{array}$$

10 $$\begin{array}{*{20}c} {mAP = \frac{\sum AP}{n}} \\ \end{array}$$

As indicated in Eqs. 7–10, TP refers to the number of correctly identified rice pest areas, FN represents the number of missed detections, and FP denotes the number of false detections. n denotes the number of categories, and the AP value is the area under the curve obtained by combining various recall (R) and precision (P) points.

## Results and analysis

### Experimental platform and parameter settings

The models are tested and trained using the Pytorch 1.8 framework on the Linux operating system. The local hardware setup features 16 GB of memory, an Intel Core i3 12,100 CPU, and a GeForce GTX 3060-12G GPU. The server is equipped with 64 GB of memory, an NVIDIA TITAN Xp 12 GB GPU, and an Intel Xeon E5-2650 v3 @ 2.30 GHz CPU. The system also includes CUDNN 8.2, a deep neural network acceleration library, and CUDA 11.2, a parallel computing framework, with Python 3.8 installed. In the training process, stochastic gradient descent (SGD) is used for optimization, with a batch size of 32, a learning rate of 0.01, and a weight decay of 0.005. The input image size is 640 × 640 pixels, momentum is set to 0.937, and the model trains for 200 epochs. To save time, model training is performed on the server and validation is done in a local environment.

Through multiple experiments, paper has confirmed that this configuration maximizes GPU computing power, greatly speeding up both model training and inference. By leveraging the rich deep learning tools and libraries provided by PyTorch and CUDA, model development and optimization can be carried out more efficiently. This experimental setup offers strong computing resources and a variety of tools for rice pest detection, enhancing the model’s performance and accuracy to better address challenges in real-world applications.

### Ablation experiment

To thoroughly evaluate the effectiveness of the RP-DETR network model presented in this paper, the paper carries out a series of ablation experiments. Based on RT-DETR RT18 as the baseline model, training was performed under the same training parameter conditions, and three improvement schemes were introduced: RepPconv-block (RPB), Gather-and-Distribute (G&D), and MPDIoU as a loss function improvement. The ablation experiment results, presented in Table 2, examine how these improvements affect the performance of the RT18 model.

**Table 2:** Ablation experiment

Code	RPB	G&D	MPDIoU	Parameter	Precision	mAP@0.5	GFLOPs/G
A				20.18 M	85.6	75.5	56.8
B	✓			14.35 M	83.6	74.5	44.6
C	✓	✓		16.77 M	86	76.7	47.8
D	✓	✓	✓	16.77 M	87	76.9	47.8

Among them, experiment A is a benchmark experiment based on the RT-DETR network structure, and experiment D is the proposed RP-DETR network model, which uses the RepPconv-block (RPB) and Gather-and-Distribute (G&D) to improve the RT-DETR model and adopts the new MPDIoU loss function. Experiments B and C are ablation experiments on the individual modules of Experiment D. The experimental results indicate that Experiment D outperforms Experiments A, B, and C significantly. The RP-DETR model presented in this paper delivers the greatest accuracy while using the least number of parameters. Experimental results demonstrate that this model can identify a broader range of pests with greater precision, all while requiring fewer device resources for rice pest detection. Table 3 compares different loss functions in this paper through experiments. By comparing the commonly used loss functions CIoU, DIoU, GIoU, and SIoU, it is demonstrated that MPDIoU has a significant effect on improving detection accuracy.

**Table 3:** Loss function ablation experiment

Loss function	Precision	mAP@0.5
SIoU	84.2	75.7
GIoU	86	76.7
CIoU	84	76.1
DIoU	86.1	76.5
MPDIoU	87	76.9

### Comparative experiment

**Fig. 7:**
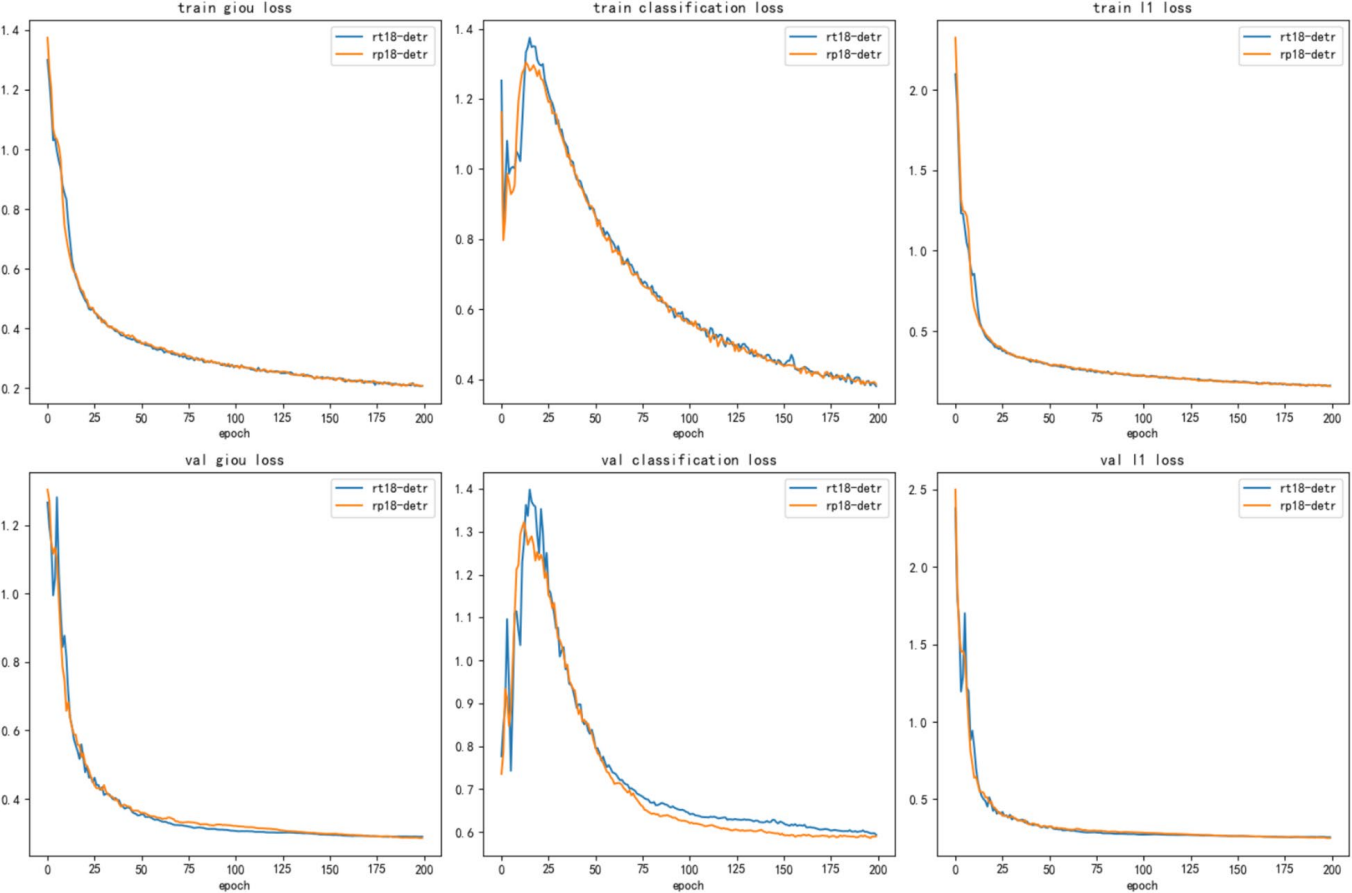
Training and testing loss for RT18-DETR and RP18-DETR

This paper evaluates the performance of the proposed model for detecting rice pests through several comparison experiments. First, a comparative analysis of the learning performance of RT18-DETR and RP18-DETR during training is shown in Fig. 7. RT18-DETR and RP18-DETR use three different losses during training and testing: giou loss, classification loss, and l1 loss, during training and testing for RT18-DETR and RP18-DETR. As can be seen from the figure, RP18-DETR does not differ greatly from RT18-DETR when l1 loss is applied, has a slight advantage over RT18-DETR when giou loss is applied, and has a significant improvement in the test fitting effect when classification loss is applied.

**Fig. 8:**
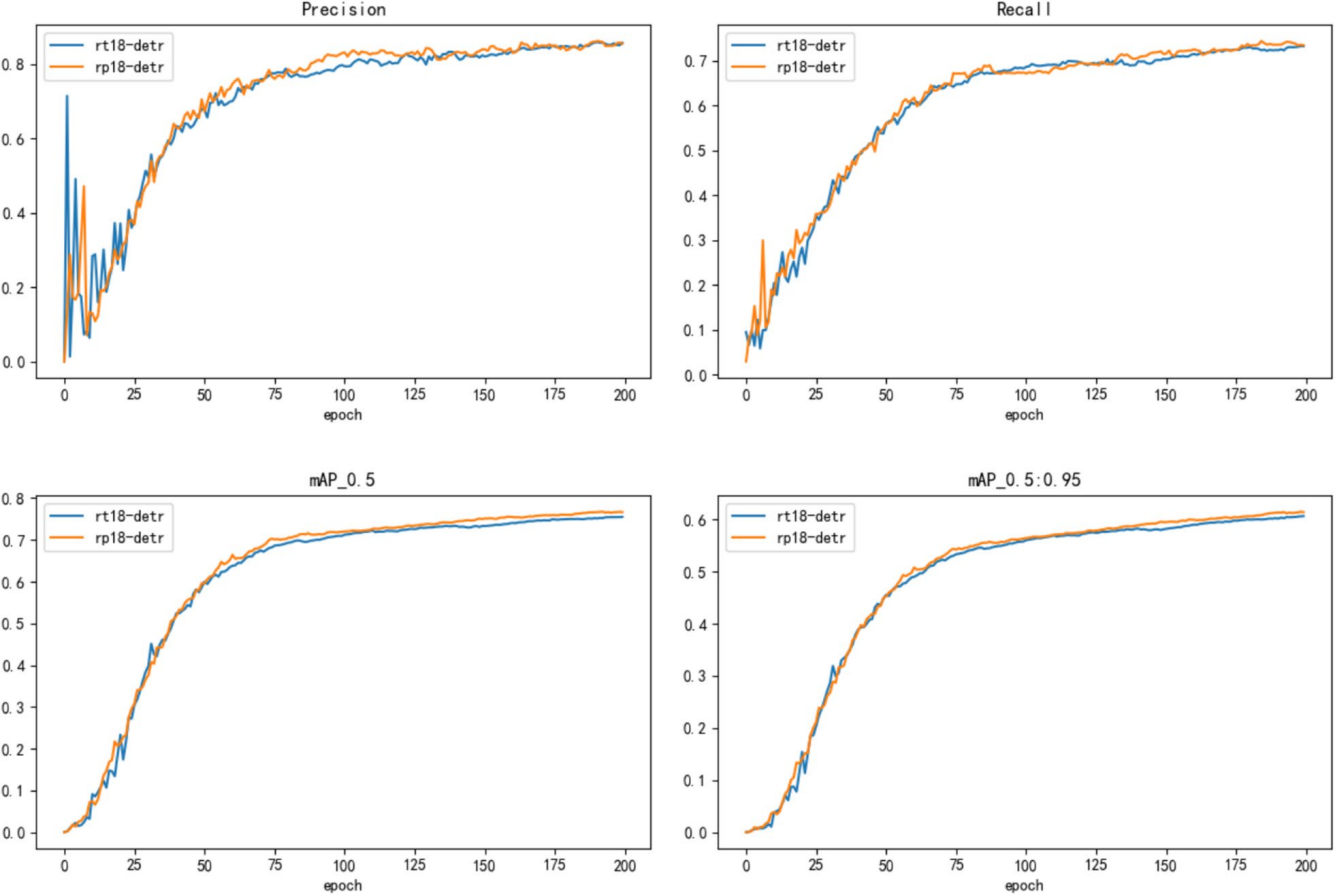
RT18-DETR and RP18-DETR training metrics

Figure 8 presents the precision curve, recall rate curve, and mAP learning curve at various IoU thresholds. The comparative experiment described above comprehensively evaluated the model’s performance on different indicators, providing sufficient support for the model’s effectiveness. Analysis of Fig. 8 shows that the learning effectiveness of the RT18-DETR and RP18-DETR models tends to slow down after about 75 epochs. The RP18-DETR model introduced in this paper outperforms the RT18-DETR model in both precision and recall, highlighting the superior performance of RP18-DETR in the rice pest detection task.

**Fig. 9:**
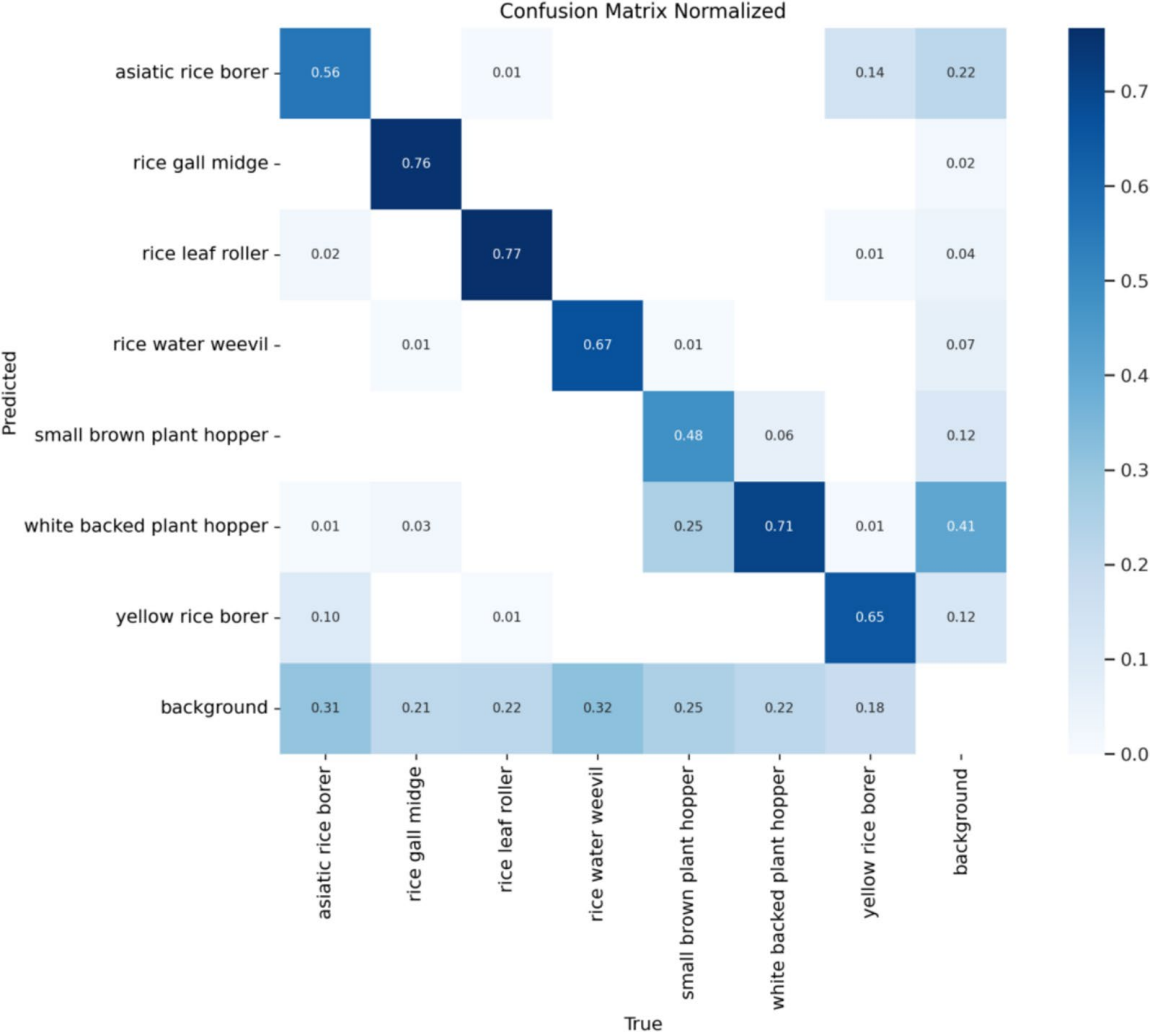
RP-DETR model confusion matrix normalization plot

Figure 9 presents the normalized confusion matrix plot for the RP-DETR model, which is a key tool in machine learning. It can help us more clearly observe the results of each classification trained by the model. The figure clearly shows that the diagonal cells are darker, indicating a higher ratio in the normalized plot, which suggests better fitting performance.

To evaluate the performance of the models at different parameter scales within the DETR framework, ResNet18 and ResNet34 are chosen as the backbone networks, and a comparative analysis of various performance metrics is conducted. The experimental results are presented in Table 4. Furthermore, this paper compares the average recognition accuracy of various pest categories between RT18-DETR and RP18-DETR, with the findings displayed in Table 5.

**Table 4:** Comparison results of different models with the same loss function

Model	FPS_bs=1_	Parameters/M	Recall/%	mAP@0.5/%	GFLOPs/G
RT18-DETR	200	20.1	73.3	75.5	56.8
RT34-DETR	95	31.4	76.7	77.3	90.6
RP18-DETR	270	16.77	73.5	76.7	47.8
RP34-DETR	98	23.3	74.1	76.9	65.4
Conditional-R50-DETR	–	44	62.2	70.9	90

**Table 5:** Comparison of the average accuracy of different rice pest damage

Classification	RT18-DETR mAP@0.5	RP18-DETR mAP@0.5	
Asiatic rice borer	52.5		56.1
Rice gall midge	94		95.1
Rice leaf roller	86.3		87.7
Rice water weevil	85.2		86.4
Small brown plant hopper	67.7		62.9
White backed plant hopper	74.5		73.7
Yellow rice borer	71.5		73

Based on Tables 4 and 5, the parameters of the RT-DETR model with ResNet18 and ResNet34 backbones, as selected in this study, surpass those of the models introduced in this paper. Among them, the RP18-DETR and RP34-DETR models have 16.5% and 25.8% fewer parameters than the original models, respectively. In terms of average accuracy, RP18-DETR is 1.2% higher than RT-DETR when the threshold is 0.5. The proposed model also shows better computational complexity. Table 4 shows that the RP18-DETR model can process 270 images per second, far exceeding the baseline model and demonstrating the results of model lightweighting.

### Model test results

**Fig. 10:**
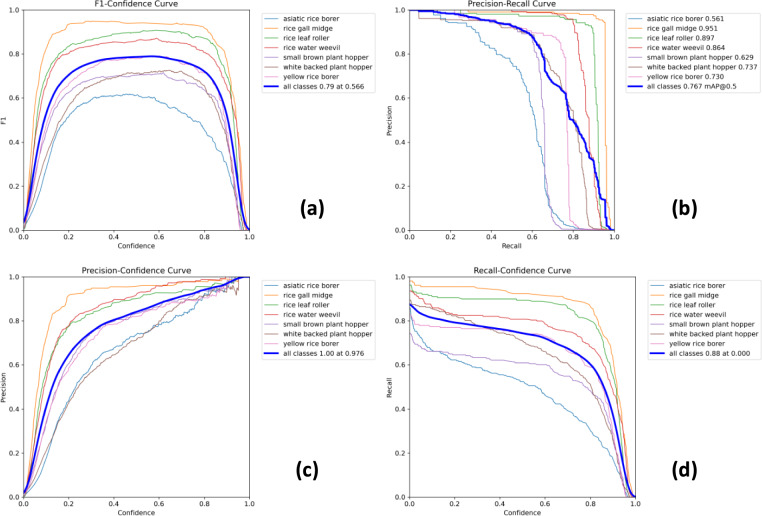
RP18-DETR model effect diagram **a** F1-Confidence Curve. **b** Precision-Recall Curve. **c** Precision-Confidence Curve. **d** Recall-Confidence Curve

In this experiment, the RP18-DETR model was used to train for rice pests. Figure 10 displays the Precision-Recall Curve, F1-Confidence Curve, Recall-Confidence Curve, and Precision-Confidence Curve throughout the model training.

In the upper left corner of Fig. 10a, the F1-Confidence curve is displayed, representing the model’s accuracy in detecting and classifying seven types of rice pests. They offer an understanding of seven distinct types of learning performance in rice insect pests. Most of these curves have a large area enclosed by the coordinate axes, and their training effects are better. The region bounded by the rice gall midge curve and the coordinate axis is the largest, resulting in the best training effect. The curves of the other three graphs also mostly exceed their diagonals, and most of the curves are close to the upper-left or upper-right corners. A larger enclosed area between the curve and the x- and y-axes indicates a better effect. The four graphs in Fig. 10 show large enclosed areas between the curve and the axes, indicating excellent effects.

**Fig. 11:**
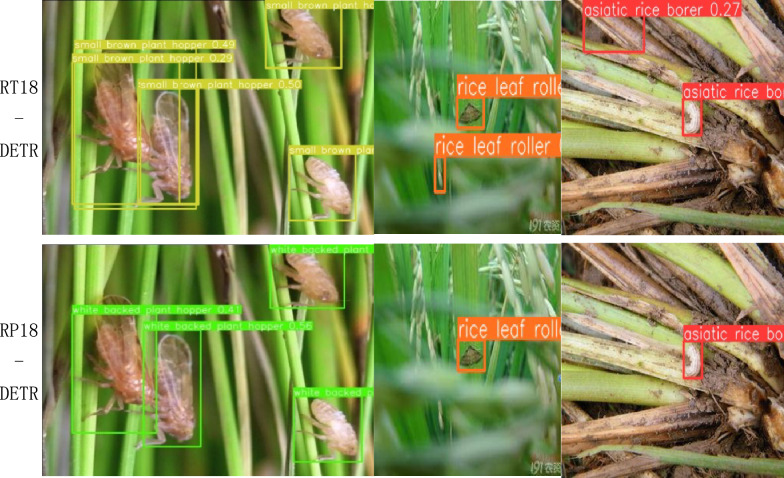
Comparison of rice pest detection results

The results of multiple image detections of rice pests using the RP18-DETR and RT18-DETR models in this experiment are shown in Fig. 11 below.

The experimental results in Fig. 11 demonstrate that the RP-DETR model introduced in this paper outperforms others in the rice pest detection task. The RT-DETR model tends to mistakenly identify the rice white-backed plant hopper as the small brown plant hopper during detection. In addition, RT-DETR has the problem of repeated detection boxes during detection, and in the detection task of the asiatic rice borer, the original model misidentified the background as the target. These results indicate that the RP-DETR model enhances detection accuracy while minimizing false positives.

## Discussion

The innovative structure and model of this research institute’s rice pest detection have achieved good results in accurately detecting different pests. However, there are still some key aspects that require further research and discussion.

The research on the collected dataset plays a crucial role in shaping both the process and the outcomes of the study. Although this study has collected numerous images from various online and offline channels and selected high-quality pest samples, which have been carefully processed and selected manually again, there may still be some biased and incomplete data images in the dataset. At the same time, the dataset does not fully cover all pest species, and samples may cause the model’s performance and effectiveness to decline in other unknown environments. Meanwhile, the dataset has been manually labeled and filtered, which may result in labeling errors or omissions due to reasons such as the filter’s fatigue, bias, and knowledge base. In future studies, the paper plans to incorporate computer programs to support manual labeling and screening, aiming to enhance the dataset’s accuracy and completeness. For instance, unsupervised learning or AI-driven recognition can be combined with manual labeling to enhance the dataset’s quality. The RP-DETR model proposed in this paper has higher accuracy than the baseline model, uses a lightweight structure, and has real-time detection. The model improves model generalization ability by fusing multi-source data sets. However, the generalization ability of the model for more rare insect pests has yet to be verified, and its adaptability to extreme weather and densely shaded environments remains to be studied in the future.

Secondly, a model’s computational efficiency and its use of resources, such as GPUs and memory, remain key factors in assessing its quality. Although the model has been improved using RepPconv-block and Gather-and-Distribute, there is still more room to continue optimizing the model, adopt newer technologies, explore ways to improve computational efficiency, enhance model accuracy, and reduce the dependence on graphics cards and memory. In subsequent research, it is planned to strengthen the model through unsupervised learning. It is also possible to try to build a more efficient intelligent rice pest detection system through hybrid architectures and other methods by tapping into the potential Mamba has shown in the field of sequence modeling. New technologies can be used to try to improve model performance, but there are also many limitations and challenges. In model adaptation, small target data may not be handled well, and there is a need for more computing resources and an increase in the size of the data set. These limitations and challenges need to be overcome in future research.

Lastly, the paper plans to apply model simplification to mobile terminals in our future work plan, monitor photos taken in the field in real time, and deploy sensing and shooting equipment in real-world fields and other environments, which will make it possible to collect more detailed and real-world image data on rice growth in the field. At the same time, in terms of the actual deployment model, this paper plans to embed the algorithm in a large-screen system to detect rice pests in real time and save the images to expand the dataset to accommodate new types of pests. The large-screen system will display detected real-world rice pests in real time. The model is still insufficient in detecting the types and quantities of rice pests and in more extreme environments compared to existing research. This paper will actively consider expanding the pest dataset in future experiments by adding normal plants, more realistic paddy field data, and more rare rice pests and more extreme environments, so that the model can be truly and effectively applied in different scenarios and applied to agricultural development.

## Conclusions

The paper proposes a highly efficient pest detection framework with an improved Transformer structure, which addresses key issues in rice pest detection. First, a high-quality dataset covering seven common types of pests was constructed, providing a solid data foundation for the deep learning model. By designing a new RepPConv-block, the model reduces both the number of parameters and information redundancy, greatly enhancing detection efficiency and real-time performance. The enhanced Gold-YOLO-Neck improves the model’s ability to fuse multi-scale features, effectively reducing false positives and false negatives under complex background interference. Additionally, the MPDIoU target regression loss function enhances the positioning accuracy for pests of varying shapes and sizes. The experimental results in this paper show that the improved framework achieves a maximum mAP of 76.9% on the self-built dataset, which is 1.2% higher than the existing method, demonstrating the model’s effectiveness and practicality. The experimental results on the self-built dataset in this paper show that the improved framework achieved a maximum mAP of 76.9%, an improvement of 1.2% over the existing method, a recall rate of 73.5%, an improvement of 0.2% over the existing model, and a reduction of 16.56% in the number of parameters compared to the existing model. The GFLOPs/G of the model is 15.84% lower than that of the existing model, requiring less time for calculation. The model is lightweight and very successful, significantly better than existing methods, and validates the effectiveness and practicality of the model.

The rice pest detection framework presented in this paper demonstrates strong performance, accuracy, and robustness, making it highly promising for practical applications. Future studies could focus on enlarging the dataset, improving the model architecture for edge computing, and exploring multimodal data fusion methods to advance the practical use and growth of pest detection technology in precision agriculture.

## Data Availability

No datasets were generated or analysed during the current study.
